# Where We Look When We Drive with or without Active Steering Wheel Control

**DOI:** 10.1371/journal.pone.0043858

**Published:** 2012-08-22

**Authors:** Franck Mars, Jordan Navarro

**Affiliations:** 1 IRCCyN (Institut de Recherche en Communication et en Cybernétique de Nantes), LUNAM Université and CNRS, Nantes, France; 2 Laboratoire d'Etude des Mécanismes Cognitifs, University Lumière Lyon 2, Lyon, France; University of Leicester, United Kingdom

## Abstract

Current theories on the role of visuomotor coordination in driving agree that active sampling of the road by the driver informs the arm-motor system in charge of performing actions on the steering wheel. Still under debate, however, is the nature of visual cues and gaze strategies used by drivers. In particular, the tangent point hypothesis, which states that drivers look at a specific point on the inside edge line, has recently become the object of controversy. An alternative hypothesis proposes that drivers orient gaze toward the desired future path, which happens to be often situated in the vicinity of the tangent point. The present study contributed to this debate through the analyses of the distribution of gaze orientation with respect to the tangent point. The results revealed that drivers sampled the roadway in the close vicinity of the tangent point rather than the tangent point proper. This supports the idea that drivers look at the boundary of a safe trajectory envelop near the inside edge line. Furthermore, the study investigated for the first time the reciprocal influence of manual control on gaze control in the context of driving. This was achieved through the comparison of gaze behavior when drivers actively steered the vehicle or when steering was performed by an automatic controller. The results showed an increase in look-ahead fixations in the direction of the bend exit and a small but consistent reduction in the time spent looking in the area of the tangent point when steering was passive. This may be the consequence of a change in the balance between cognitive and sensorimotor anticipatory gaze strategies. It might also reflect bidirectional coordination control between the eye and arm-motor systems, which goes beyond the common assumption that the eyes lead the hands when driving.

## Introduction

Eye-hand coordination is important in many tasks that we perform on a daily basis, such as reaching to grasp, drawing or playing ball games [1]–[4]. Typically, the eyes are proactive, seeking out useful information in the moment leading up to the hand movement. The goal may be to identify and provide knowledge about the fixated object, but extra-retinal information (ocular proprioception and efference copy of motor commands sent to the eye muscles) can also be used to guide distal motor systems, such as the hand that reaches out or the foot that steps forward. Driving a car relies on such visuomotor coordination. In their seminal paper, Land and Lee [5] showed a strong coupling between gaze and steering control, with the steering wheel angle and the gaze angle showing the same dynamics, with a time-lag of about 0.8 s between eye and hand movements.

Land and Lee [5] also observed that drivers spend a significant amount of time looking close to the tangent point (TP); that is to say, the point where the direction of the inside edge line seems to reverse from the driver's viewpoint. The authors proposed that this particular road feature is used because it can be easily isolated and tracked in the visual scene and also because the angle between the direction of heading and the direction of the TP has a simple geometrical relationship with the road curvature. Hence, looking at the TP may be an efficient way of “reading” the road curvature at the sensorimotor level. The so-called TP strategy hypothesis has been the subject of criticism. Some authors [6], [7] have favored an alternative hypothesis, which states that when driving, one looks at the points in the world through which one wishes to pass. According to this hypothesis, fixations on or near the TP result from trying to take a trajectory that cuts the bend [8]. Thus, although it has been repeatedly demonstrated that drivers spend between 50% and 75% of the time looking in the close vicinity of the TP when negotiating bends [9]–[12], the exact nature of gaze-sampling strategies is still a matter of debate.

Preventing eye-steering coordination by means of a static fixation target negatively impacts various indicators of driving performance [7], [13]. On the other hand, enforcing this coordination by instructing drivers to continuously use the TP strategy improves steering stability [9]. Mars [14] has demonstrated that tracking any visual feature by following the dynamics of the TP, not necessarily the TP proper, yields the same benefits. All these observations support the idea that eye movements guide steering actions in a similar way to a hand tracking a moving target when the eyes produce smooth pursuit of the object. In other words, the ocular control system feeds into the manual control system to improve its performance. Information about eye movements may be sent to the arm-motor system through ocular muscles proprioception, efference copy of oculomotor command and predictive estimation using an internal model of the eyes' dynamics [15], [16].

Eye-steering coordination in driving has always been considered as unidirectional, i.e. the eyes lead the arms. The reciprocal influence of steering actions on gaze behavior has never been investigated, and yet there is a strong indication of bidirectional coupling between the eyes and the hands during manipulative, reaching and tracking tasks. It has shown for instance that the oculomotor system has access to an efferent copy of arm- motor commands during the smooth pursuit of a visual target [17]. Reina and Schwartz [18] also demonstrated that a visuomotor illusion of hand movement could influence gaze control during closed-loop drawing. Vercher and colleagues [19], [20] proposed a model that assumes the characteristics of hand movements are stored and considered by the eye. Both motor systems are independent, but exchange information in the form of sensory and motor signals, possibly mediated by a forward model of the arm [21]. Hence, it is reasonable to hypothesize that the visuomotor control of steering is a dynamic control loop in which the execution of action determines gaze sampling strategies as much as eye movements guide steering actions. On the other hand, it has been demonstrated that, during action observation, humans instinctively produce anticipatory eye movements equivalent to those used in action [22]. If this applies to the visual control of steering, drivers may activate very similar eye motor programs when moving the steering wheel themselves and when observing another agent doing it.

Based on these considerations, the objective of the present study was two-fold. First, we aimed to contribute to the understanding of the so-called TP steering strategy. This was done by quantifying how glances are distributed in space in relation to the TP. The second objective was to determine whether the active performance of steering movements during driving influences the visual anticipatory tracking of the road. To this end, the distribution of gaze fixations when drivers negotiated a series of bends was scrutinized in two conditions. In one condition, the participants actively performed arm movements to steer the vehicle. In the other, the hands were moved passively by the steering wheel, which was controlled by lane-following automation.

## Methods

### Participants

Seventeen healthy volunteers with normal or corrected to normal visual acuity participated in the study. In order to obtain a good calibration of the gaze-tracker, astigmatic subjects and subjects wearing glasses were not eligible to participate. Mean age and standard deviation was 27.1±7.4. They had 7.8±4.5 years of driving experience with an average estimated mileage of 9617 km per year. None of them suffered from simulator sickness. All participants gave written informed consent. The experiment was performed with approval by the CNRS operational ethics committee (Comité Opérationnel d'Ethique, Copé) and in accordance with the Declaration of Helsinki.

### Material and procedure

The experiment was conducted using the fixed-base SIM^2^ simulator [23], which included an adjustable seat, a steering wheel with force feedback, a gear lever, clutch, accelerator and brake pedals, and a speedometer. The visual environment was retroprojected onto a large translucent screen, viewed from a distance of about 2 m. The visual angle of the stimulus was approximately 62×51 degrees. The graphic database reproduced a 3.4 km long two-lane main road with bends of various length and curvatures. The driving lane was 3.3 m wide and delineated with a broken centerline and a continuous edge line. All data coming from the simulator, including speed, lateral position of the car and the coordinates of the tangent point in the visual scene, was recorded with a frequency of 5 Hz.

The driver's gaze was recorded by means of the IviewX head-mounted gaze tracker (Sensomotoric Instruments), which sampled eye movements at 50 Hz. The gaze-tracker was coupled with a head-tracking device in order to compensate for head movements and compute gaze position in the reference frame of the screen. Using a 13 points calibration procedure, gaze position accuracy was about 0.5°.

After the participants were installed in the simulator and eye tracker calibration carried out, they were invited to start the simulator and drive twice round the whole track for training purposes. Then, the experiment proper began. A repeated measures design was used. Four laps were completed with the participants steering the vehicle themselves (active steering) and four laps with an automatic controller performing the task (passive steering). The automatic steering system was adapted from the proportional controller presented by Chaib et al. [24]. It continuously computed a desired steering angle as a function of the heading error of the vehicle relative to the axis of the road and the vehicle lateral position. An actuator installed on the vehicle's steering column and driven by the control law ensured that the vehicle followed the desired steering angle. Whenever an external torque was applied on the steering wheel, the automation switched off. The torque threshold for system disengagement was low enough that the participants strictly needed to follow the steering wheel movements without active participation. Whenever switching off occurred, the trial was terminated and ran again later, but this barely happened.

The order of presentation of the active and passive conditions was counterbalanced. In both cases, the participants were instructed to constantly keep their hands on the steering wheel in a standard 10-to-2 position, to comply with the speed limits and to keep the vehicle within the lane boundaries. They were also informed that they should be ready to skirt around unannounced obstacles at all times. Obstacles actually appeared four times during the 2nd, 4th, 5th and 7th laps, in one of two positions on the track. Hence, they were relatively unpredictable and appeared frequently enough to ensure that drivers knew they were in charge of steering and could not rely on the automation in the passive steering condition.

### Data analysis

Data from the driving simulator, including the coordinates of the TP, the lateral position of the vehicle and its speed, were synchronized with the data from the gaze-tracker. Each gaze coordinate was then converted into an angular deviation from the tangent point (in degrees). Data obtained in identical conditions (active or passive steering) were regrouped. The track was divided into 18 sections. Data obtained in straight lines, in short curved sections (less than 80 m in length) and in the two bends where obstacle skirting occurred were discarded. A set of seven different bends remained. Table 1 indicates their direction, length and mean radius.

**Table 1:** Characteristics and results for individual bends.

Bend identifier	Direction	Length	Mean radius	% TP active	% TP passive
B1	L	140	500	73.9	65.5
B2	R	180	440	76.3	70.4
B3	L	130	231	69.6	68.4
B4	L	105	127	74.2	76.6
B5	R	85	100	67.5	62.8
B6	L	120	400	68.1	61.8
B7	L	100	130	73.7	66.2
Total		860	305	72.2	67.5

For each bend, direction (L: left, R: right), length (m), mean radius of curvature (m), and proportion of time spent in the tangent point area in the active and passive steering condition.

**Figure 1:**
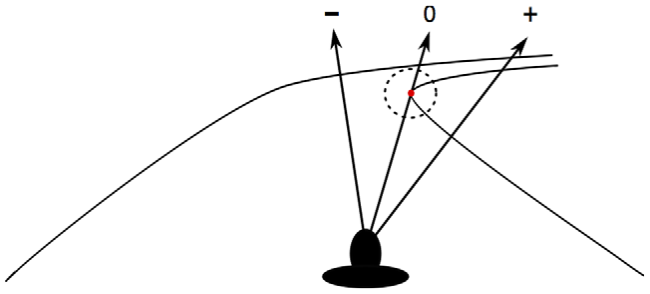
Schematic representation of data analysis methods. The TP (red dot), the 5° area around the TP (dotted circle) and the sign convention for the horizontal gaze deviations from the TP (arrows) are represented.

The percentage of time spent by drivers looking in a region of 5° around the TP was computed as a global indicator of gaze positioning (Figure 1). The difference between the active and passive steering conditions was examined using a paired t-test. In order to reach a more detailed understanding of the distribution of gaze relative to the TP, the proportion of gaze points in intervals of 1° of horizontal angular deviation from the TP were computed. The signs of measurements obtained in left bends were changed so that a negative value represented a deviation of gaze toward the road centerline, whereas a positive value represented a deviation of gaze in the direction of the bend exit, independently of the bend direction (Figure 1). All gaze points that deviated more than 15° in one direction or another were binned in two extreme classes. Two-way repeated measures analyses of variance (ANOVA) with the experimental condition (active vs. passive steering) and the angular deviation from the tangent point (32 levels) as independent variables were performed on the obtained data. Bonferroni corrections were used for post-hoc analyses. The statistical significance level α was set at 0.05. All tests of significance were supplemented by a variant of Bayesian statistical inference (fiducial inference: see [25], [26]), which allowed us to draw conclusions on the population effect size (δ) as a function of the observed effect (d), sample size and variability. This method considers test power and goes beyond a conclusion in sole terms of non-null effects. A conclusion such as δ>a (short for P(δ)>a = γ) should be read as “there is a high probability (guarantee γ) that the population effect is larger than the value a”. All fiducial conclusions are given with the guarantee γ = .90.

## Results

When actively steering the car, drivers spent 72.2% of the time looking in the region of 5° around the TP. In the passive condition, this proportion was significantly reduced to 67.5% (t_16_ = 3.37, p<.005, δ>2.88). As seen in table 1, the reduction was observed in all bends, except B4.

**Figure 2:**
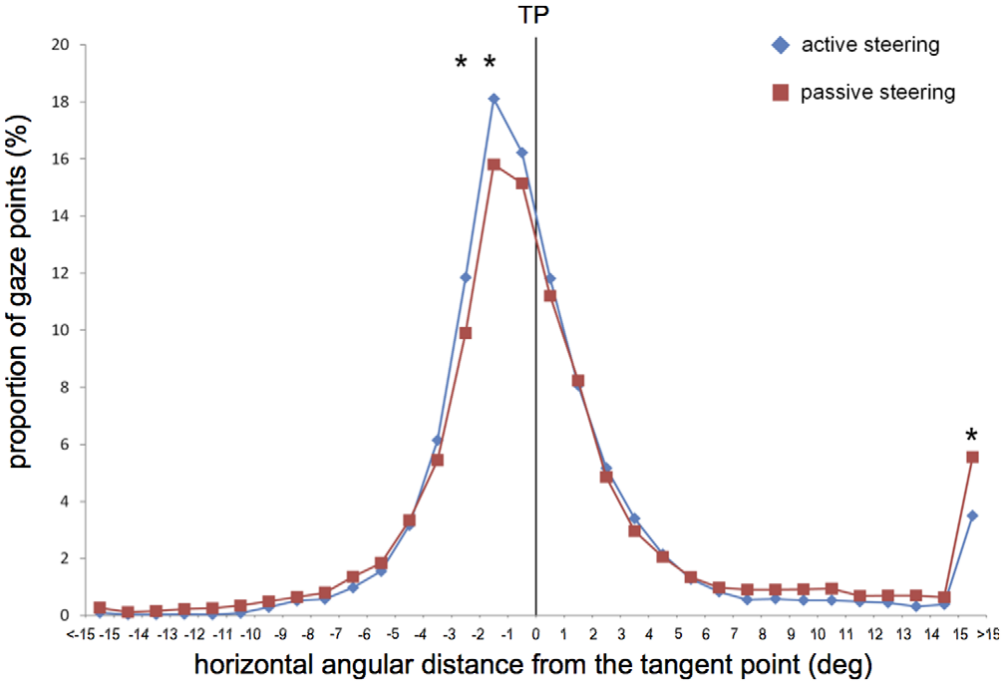
Distribution of gaze points as a function of horizontal angular deviation from the tangent point. A positive value on the x axis represents a gaze deviation in the direction of the bend exit (to the left for left bends, to the right for right bends). Asterisks indicate significant differences between active and passive conditions.

Figure 2 represents the distribution of gaze points as a function of the horizontal angular deviation from the tangent point in the active and passive steering conditions. It can be observed that the peak of the distribution deviates from the TP in both conditions, with a high concentration of gaze points in a 2° visual angle adjacent to the TP and in the direction of the lane center (34.3% in the active condition, 30.9% in the passive condition). The ANOVA performed on the data showed a non-significant main effect of the experimental condition (F(1,16) = 1, p = 0.33), a significant effect of horizontal angular deviation from the TP (F(31,496) = 87.25, p<.001) and a significant interaction between both variables (F(31,496) = 6.41, p<.001). Post-hoc tests confirmed the asymmetry of the distribution. When comparing the percentage of gaze points on each side of the TP ([−1°,0°] vs. [0°,1°]), significantly more fixations were oriented toward the lane center than toward the bend exit. This was observed in the two experimental conditions (active: d = 4.41, δ>3.12, p<.001; passive: d = 3.94, δ>2.41, p<.005). Post-hoc tests also revealed that the effect of active vs. passive steering was significant for three angular deviations only. During active steering, a significantly higher proportion of gaze points was observed for angular deviations between 1° and 3° from the TP in the direction of the lane center ([−3°,−2°]: d = 1.95, δ>1.26, p<.001; [−2°,−1°]: d = 2.31, δ>1.52, p<.001). During passive steering, a significantly higher proportion of gaze points was observed for angular deviation above 15° in the direction of the bend exit (d = −2.05, δ<−1.27, p<.001).

The mean speeds of the vehicle in the active and passive conditions did not differ significantly (78.1 and 77 km/h respectively, t_16_ = 0.81, p = .43, |δ|<2.9). The automatic steering system adopted trajectory profiles that were similar to those of the drivers, in which there was cutting of corners in bends. This translated as a significant deviation of the mean lateral position of the car from the lane centre in the direction of the inside edge line in both conditions (active: t_16_ = 8.86, p<.001, d = 0.4, δ>0.34 m; passive: t_16_ = 13.35, p<.001, d = 0.24, δ>0.21 m).

## Discussion

This study investigated whether the active performance of steering movements influences anticipatory gaze control during driving. Although it is widely accepted that the eyes lead the arms when driving, the reciprocal influence of active arm motion on gaze control has been evaluated here for the first time. The results show that the distribution of gaze orientation was in large part similar in both conditions, but a small reduction in the proportion of glances directed to the TP area was observed during passive steering. A detailed inspection of the data revealed that this reduction was mostly limited to a narrow visual angle, 2° wide. This corresponded to the part of the visual scene where the highest proportion of glances was directed, that is to say, on the roadway with a small angular deviation from the direction of the TP. On the other hand, when steering was passive, the drivers looked more often beyond the TP area, in the direction of the bend exit.

When Land and Lee [5] first observed that drivers spent a large amount of time looking in the area of the TP when driving along a winding road, they proposed an elegant and economical solution to the question of where drivers look in order to preview the road and steer the car accordingly. If there is a salient visual feature in the environment that moves proportionally to the road curvature, the central nervous system may learn to use this feature as a target for the eyes. In turn, extra-retinal information arising from the tracking of this target may provide some input to the arm-motor system in charge of steering the car. The TP hypothesis has received a great deal of attention over the years, recently becoming the object of controversy. Recent publications that have investigated gaze strategies when driving offer either replications or variations of Land and Lee's observations [9]–[12], [27], [28], as well as more or less assured rebuttals [7], [8], [29]. According to the alternative hypothesis, drivers look in the direction they want to steer, which often happens to be near the TP. This is in accordance with the hypothesis that the curvature of the projected retinal flow along the future path provides some information about steering errors [6], [30], [31]. Our results do not fully support either hypothesis. A high concentration of gaze points in a small area around the TP was observed, which at first sight may be interpreted as a support for the TP hypothesis. However, the peak of the distribution of gaze orientation was not centered on the direction of the TP. The largest proportion of glances deviated from the TP in the direction of the lane centre, which demonstrates that drivers mainly sampled the roadway close to the inside edge line rather than the edge line proper. One could argue that looking approximately 1 degree from the TP is effectively looking at it as it falls into foveal vision. Still, the observed error relative to the TP was not random. It was biased in the direction of the roadway whatever the direction of the bend. The hypothesis according to which drivers lock their gaze at the TP to monitor the changes in road curvature does not predict such a constant error. Neither do our results support the idea that drivers look at the future path, at least not in the way it is traditionally considered. Considering the mean bend radius (305 m) and the mean lateral position of the car (0.4 m on the inside of the lane center), the angular deviation from the TP when looking at the line of travel would be 2.6 degrees. We observed that the peak of the gaze distribution was around 1 degree from the TP, which is much closer to the edge line. In terms of distance, it corresponds to a deviation of 48 cm from the TP. Given the width of an average car, this is approximately where the inner wheel would pass over the roadway.

These observations lead to a new hypothesis on the functional role of the TP and road sampling for steering control. They support the idea that drivers track the TP to stabilize the eyes close to the edge line in a position on the roadway that they do not want to cross. In other words, visual sampling in the TP area may serve as determining a safety line that delimits an acceptable trajectory envelope. Thus, the TP is a salient visual cue in the road scene that is used to guide steering, but the final gaze position may not be the TP proper. As such, the TP may be considered as a useful dynamic spatial reference when analyzing gaze strategies during driving or as an approximate input to computational driver models [32]–[34], even though it is not exactly fixated.

In the passive steering condition, the participants did not need to perform actions on the steering wheel to remain within the road boundaries. However, the experiment was designed in such a way that drivers could not afford to disengage from monitoring the road ahead. Indeed, the drivers were instructed that they should keep their hands on the steering wheel at all times in case they needed to regain control of steering. Actually, they had to perform skirting maneuvers twice. Thus, they were put in situations in which they needed to constantly anticipate changes in road curvature. The results confirmed that visual anticipation was preserved and that the participants kept on looking in the area of the TP for the most part. However, it appears that more glances were directed to the far distance. This corresponds to an increase of “look-ahead fixations”, i.e. far anticipatory glances which are not related to the current task but allow to plan future actions in advance [35]. In the context of driving, look-ahead fixations may serve to take into account contextual information at the tactical level. For instance, a bend of a given radius and length may be negotiated at different speeds depending on whether it is succeeded by a second sharp bend or a straight line. The detection of oncoming traffic may have similar effects on anticipatory speed and steering regulation. Both look-ahead fixations and TP tracking may be considered as anticipatory gaze behavior, in contrast with short-term corrections of lateral position errors, which presumably rely on peripheral vision [36]–[39]. Look-ahead fixations, like glances to the TP area, may even serve in part to anticipate changes in road curvature, but it can be hypothesized that the two are different in nature. The primary function of looking at the TP area may be to provide frequent input to the nervous system in charge of visuomotor coordination (i.e. what can be considered as a sensorimotor “reading” of the road curvature), whereas look-ahead fixations bring knowledge about road features and are processed by higher-order cognitive functions.

Thus, it appears that during passive steering, drivers did not drastically change the way they sampled the road scene, but they favored looking far-ahead over tracking the TP by comparison to active steering. This may be interpreted as the consequence of a decision by drivers to withdraw from the steering task to some extent in spite of the instructions not to do so. As the need for visuomotor coordination was not as strong as during active steering, the participants may have chosen, not necessarily consciously, to look for information further down the road. In other words, it is very plausible that obstacle avoidance became more a priority than monitoring the changes in road curvature. The drivers may have searched to detect more in advance the occasional obstacles.

The increased number of look-ahead fixations in the passive condition may be explained only by cognitive factors. The question remains to determine whether the active control of arm movements emphasizes the sampling of the road in the TP area at the sensorimotor level. It has been proposed that a reciprocal exchange of information exists between the eye and arm-motor systems, particularly the fact that efference copy of the arm-motor command is used to enhance coordination between the eyes and the arm [40]. Vercher and colleagues [19]–[20] proposed a physiologically grounded model of eye-hand coordination in which a coordination control system receives signals from the eye and arm-motor systems. Coordination control is achieved in the cerebellum through the exchange of non-visual signals (proprioception and efference copy) between the arm-motor system and the oculomotor system, rather than by common commands addressed simultaneously to the two systems. Efference copy from the moved arm seems to plays a crucial role in timing (synchrony between arm and eye motion onsets), while arm muscle proprioception is needed for spatial aspects (accuracy). Transposed to eye-steering coordination during driving, this model would predict changes in the dynamics of the pursuit of any visual target used for previewing road curvature when the active control of the steering wheel is suppressed. The low acquisition frequency of the simulator data in our experiment, including the computation of the tangent point position, meant that it was not possible to perform in-depth analyses of the spatiotemporal patterns of eye-hand coordination. Hence, it cannot be concluded from the present data that the observed increase in eye orientation directed close to the TP is the result of an improvement in the timing of eye and hand movements. It might only be that active control steering called for more glances in the TP area in order to provide additional input to eye-steering coordination. This should be investigated in future studies using higher frequency acquisition.

In summary, this study examined gaze sampling strategies when negotiating bends in the road. The results lead to a new hypothesis according to which drivers look in the vicinity of the TP for the boundary of an acceptable trajectory envelope they do not want to cross. It also demonstrated for the first time that the active performance of steering wheel movements yields fewer look-ahead fixations and more glances to the part of the roadway that subserves visuomotor coordination when compared with passive steering.
